# 

**Published:** 1901-12

**Authors:** 


### INDEX TO VOLUME XXII.

A.

Abscess, a valuable new device to relieve an, 378
ABSTRACTS AND TRANSLATIONS:
After-results of treatment of caries by geranium-formol, 26
Death under nitrous oxide, 167
Gelatin as a hæmostatic, 101
Medicine of the nineteenth century, 161
Spinal anæsthesia, 93
The comparative germicidal action of some disinfectants, 97
The mosquito-malarial fever, 95
The recent death under nitrous oxide gas, 89
Unqualified dental practice, 164
Academy of Stomatology, 44, 111, 168, 264, 336, 420, 502, 711
resolutions of respect to Henry H. Burchard, 137
Actinomycosis, 165, 231, 402
Alcohol as an antiseptic, 434
Allan, George S., boxings, 73
Alloy of gold and platinum for backings, seamless crowns, and caps, 343
Aluminum, process of manufacture of, 675
chloride of silver a solder for, 676
versus gold for clasps, 435
Alveolaris, syphilitic localosis, 232
Amalgam, experiments upon, as a filling material, 13
to remove fillings of, 255
American Academy of Dental Science, 53, 185, 233, 407, 570, 755
citizenship, 53
American Dental Society of Europe, 896
Medical Association, Section on Stomatology, 856
address of the chairman of, 473
editorial on, 502
meeting of, 370, 549, 639, 702
resolutions congratulating Dr. Marshall, 560
Anæsthesia, local, without risk or danger, 812
prevention of nausea and vomiting during, 637
Anæsthetics, deaths in England under, 198
Anderson, Martha, methods in the preparation of teeth, 625
Andrews, R. R., address of the chairman of the Section on Stomatology of the American Medical Association, 473
Antisepsis, oral, in relation to preventive medicine, 687
Antrum, testing for disease of, with a tuning-fork, 518
Apparatus, simple electro-gold-plating, 730
Appliance, new retaining, 417
some new, 377
Army, dental surgeons in, 197, 210
United States, dental service in, 477
Arrington, B. F., free use of tooth-brushes essential for healthful preservation of teeth and gums, 225
much bothering about a non-essential for success in practice, 78

Assistant, the use of an, 75
  discussion on, 104
Association, microbic, 520
Autointoxication, interstitial gingivitis as a prominent obvious early symptom of, 746

B.

Backings, alloy of gold and platinum for, 343
  method of fitting, 343
Bacteria, the tongue as a breeding-place for, 832
Bacteriology, 632
Bascom, H. S., case of irregularity of superior cuspid, 17
Beers, W. George, obituary, 135
  monument to the late (editorial), 124
Beeswax, to test, 727
Benton, J. H., prophylaxis, 693
Bibliography:
  Annual and Analytical Encyclopædia of Medicine. By Charles E. de M. Sajous, 661
  A Practical Treatise on Artificial Crown- and Bridge-Work and Porcelain Dental Art. By George Evans, 128
  A Practical Treatise on Materia Medica and Therapeutics. By J. V. Shoemaker, 125
  Dental Anatomy Note-Book. By Douglas Gabell, 199
  Dental Electricity. By Levitt E. Custer, 514
  Irregularities of the Teeth and their Treatment. By Eugene S. Talbot, 804
  New Modes of Thought based upon the New Materialism and the New Pantheism. By C. T. Stockwell, 897
  Oral Pathology and Practice. By W. C. Barrett, 598
  Our Teeth and How to take Care of Them. By Victor C. Bell, 430
Bibliography:
  Pathology of the Teeth. By Carl Wedl, 280
  Poems on the Farm, and other Poems. By Charles Nelson Johnson, 430
  Principles and Practice of Filling Teeth. By C. N. Johnson, 130
  The American Text-Book of Operative Dentistry. By E. C. Kirk, 59
  Treatment of Malocclusion of the Teeth and Fractures of the Maxillæ, review of. By Edward H. Angle, 62
  X-Light Apparatus. By William Rollins, 200
Bierworth, J. C., uric acid in its relation to disease, with special reference to the Diseases of the Oral Cavity, 534
Biology, investigations in, 478
Bite, unilateral jumping the, 315
Blindness, gold, its treatment, 284
Board of Dental Examiners of Pennsylvania, 715
  the Army Dental Examining, 274, 663
Boards, foreign advisory, 785
  of dental examiners, revenue for conducting the work of the, 495, 496
Bogue, E. A., impressions of the International Dental Congress, 218
Bogue, Frederick L., open letter to, 283
  relative effect of a common environment upon enamel, 293
Boil, curing a, 812
Boxings, 73
Bridge, a single support for, 258
  breaking away of a, 259
  sacrificing teeth for a, 259, 262
Bridges, fixed, to repair facing on, 434

Bridges, mounting, 728
setting, with gutta-percha instead of cement, 729
use of Logan crowns as piers in, 669
Brockway, A. H., the use of an assistant, 75

C.

Cancer, starving a, 263
of the tongue, distinguishing features of, 392
Capillarity in treatment of root-canals, 40
Carbonic acid in dental practice, 388
Carborundum, process of the manufacture of, 677
uses of, 679
Caries, after-results of treatment of, by geranium-formol, 26
the saliva a protection afforded by nature against, 696
Carpenter, George T., simple gingivitis, its etiology and treatment, 836
Carr, William, the appointment of State boards of medical and dental examiners, 480
Cases, full upper, how to lighten, with side-blocks, 432
Cement, a durable, 351
Central Dental Association of New Jersey, 671
Chilcott, L. S., the use of silver-foil in dentistry, 690
Chittenden, Charles C., the dental college standard, 485
Chloretone as an hypnotic and local anæsthetic, 383
Chupein, Dr. Theodore F., obituary, 352
resolutions of respect to, 603, 726
Clamps, dam, to secure on conically shaped teeth, 813
Clasps, aluminum versus gold for, 435
Classes, the graduating, of 1901, 278
Cleft-palate, operating for, in infancy, 221
Cocaine solution for hypersensitive dentine, 731
Collaborator, an additional, 279
Colorado State Board of Dental Examiners, 815
State Dental Association, 212, 443, 816
Commissions for research work, State societies should establish, 474
Committee, Foreign Relations, Report of, 206
Congress, the Third Pan-American Medical, 69
Conventions at Milwaukee, 655
Crown- and Bridge-Work, Artificial, A Practical Treatise on, 128
Crown, an improved, 379
Crowns, artificial, the construction of, 302
Logan banded, 284
Logan, the use of, as piers in bridge-work, 669
seamless, alloy of gold and platinum for, 343
Crowns and bridges, Dr. W. S. Davenport's system of, 223
fixed, to repair facings on, 434
mounting, 728
Cuba, some considerations of the dental outlook in, 307
Cube of glass for cement mixing, 560
CURRENT NEWS:
American Medical Association, Section on Stomatology, 370
Annual meeting of the Mississippi Valley Medical Association, 672
Board of Dental Examiners of Pennsylvania, 815
Central Dental Association of New Jersey, 671
Colorado State Board of Dental Examiners, 815
State Dental Association, 212, 443, 816

CURRENT NEWS:
Communication from the Foreign Relations Committee, 286
Dental Commissioners of Connecticut, 733
surgeons in the army, 210
Foreign Relations Committee of the National Association of Dental Faculties, 67, 355
Galveston Dental Society, 71
Harvard Dental Alumni Association, 733
Odontological Society, 211
Illinois State Dental Society, 372, 671
Institute of Dental Pedagogics, 814
Jefferson County Dental Society, 212
Massachusetts Dental Society, 441
Mississippi Valley Medical Association, 816
National Association of Dental Examiners, 443, 524, 732
Dental Association, 524, 735
New England Association of Dental Examiners, 444
New Jersey State Dental Society, 72, 442, 734
New York Odontological Society, 140
State Dental Society, 210
North Carolina State Board of Dental Examiners, 443
Northeastern Dental Association, 734
Notice, 371
Pennsylvania Board of Dental Examiners, 211, 441
State Dental Society, 815
Programme of Rochester Dental Society, 72
Rhode Island Dental Society, Resolution, 140

CURRENT NEWS:
South Dakota State Dental Society, 444
Southern California Dental Association, 734
Southern Dental Society of New Jersey, 211
Tennessee Dental Association, 442
Texas State Dental Association, 670
The Fifth District Dental Society, 292
The National Meeting at Milwaukee—Special Notice, 523
The Third Pan-American Dental Congress, 69
Tri-State Dental Association, 292, 372
a legal notice, 209
Curry, J. C., quack dentistry, 502

D.

Danforth, Guy R., the duty of young men to the profession, 150
Darby, E. T., instrument handles, 712
Davenport, A. F., stray thoughts on dental hygiene, 22
Davenport, W. S., system of crowns and bridges, 223
Deaths in England under anæsthetics, 198
Decker, W. E., a case of orthodontia diagnosed and progress watched by the X-ray, 146
Degeneracy of the pulp, symposium on, 545
Dental Commissioners of Connecticut, 733
decay, non-specificity of micro-organisms in, 317
degrees, fraudulent, 770
*Digest* and the "Faculties," 595
practice, instrumental precision in, 1
unqualified, 164

Dentine, a contribution to the sterilization of, in living and dead teeth, 841
An Attempt to Explain the Sensitiveness of, 321
hypersensitive, cocaine solution for, 731
sensitive, obtunded by ethyl chloride, 645
obtunded by intense heat or cold, 646
Dentistry, is, overcrowded? 270
the makers of, 755
wherein does, differ from trade? 796
Dentists, an argument for medical education of, 149
Dentures, refitting with oxyphosphate of copper, 520
rubber and celluloid, replacing old rubber with new in, 517
weighting full lower, 432
Diathesis, uric acid, 433
Dies, a new method of making, 351
Discussions:
A case of orthodontia diagnosed and progress watched by the X-ray, 185
A case of swallowing a tooth, 407
A few cases of replantation, 326
A unique method of tightening or inserting lower front teeth, 253
Boxings, 105, 408
Case of irregularity of superior cuspid, 32
Degeneracy of the pulp, 639
Dental education, 881
Development of the maxillary sinus, 264
Impressions of the International Dental Congress, 258
Infectious diseases, 706
Instrumental precision in dental practice, 168
Instrument handles, 715
Discussions:
Malignant growths in the mouth, incidental to general practice, 392
Military dental practice: its modifications and limitations, 856
Oral prophylaxis, 850
Periods of stress and their dental marks, 702
Prophylaxis in dentistry, 44, 233
Quack dentistry, 504
Relative effect of a common environment upon enamel, 329
Some æsthetic considerations in the treatment of the teeth in the incisal region, 756
Some new appliances, 413
State dental examining boards and their questions, 111
Stray thoughts on dental hygiene, 34
Symposium on State boards of dental examiners in their relation to the profession and the colonies, 549
The condition of the teeth of children in the public schools, 570
The construction of artificial crowns, 336
The treatment of certain oral expressions commonly designated pyorrhœa alveolaris, 647
of unfilled root-canals, 37
The use of an assistant, 104
of silver-foil in dentistry, 763
Uric acid in its relation to disease, with special reference to the diseases of the oral cavity, 563
Diseases, infectious, 686
Disinfectants, the comparative germicidal action of some, 97

District Dental Societies of New York, Union Meeting of Sixth, Seventh, and Eighth, 735
Dogs, pyorrhœa alveolaris in, 669
Domestic Correspondence:
  Circular from the War Department, 282
  Open letter to Dr. Frederick L. Bogue, editor The New York Institute of Stomatology, 283
  Reply to Dr. J. Morgan Howe, 65
    *Items of Interest*, 203
  The Army Dental Examining Board, 663
Drug, possibly a valuable, 433
Dummies, a new method of making, 433
Dunning, Edwin James, biographical sketch of, 808

E.

Eames, George F., the treatment of certain oral expressions commonly designated pyorrhœa alveolaris, 605
Editor, the, was tired, 121
Editorial:
  American Dental Society of Europe, 896
  American Medical Association, Section on Stomatology, 502
  An additional collaborator, 279
  An advance movement, 192
  Death of William Henry Morgan, D.D.S., 429
  Deaths in England under anæsthetics, 198
  Dental surgeons in the army, 197
  Is dentistry overcrowded? 270
  Is the result worthy the effort? 346
  Monument to the late Dr. Beers, 124
  Professor W. D. Miller, 802
  The Army Examining Board, 274
Editorial:
  The beginning of a new volume, 57
  The convention period, 510
  The conventions at Milwaukee, 655
  The *Dental Digest* and the "Faculties," 595
  The editor was tired, 121
  The essentials in teaching, 193
  The first year of the new century, 891
  The graduating classes of 1901, 278
  The man with one paper, 721
  The twentieth century, 55
  United States Consul Worman and his work, 893
  What does it mean? 800
  What should be next in order? 426
  Wherein does dentistry differ from trade? 796
Education, dental, 473, 878
Educational Affairs, American, in Europe, 774
Electrical current, control of, in plating, 531
Electricity as applied to chemical and metallurgical processes, 673
Electro-metallurgy, depositing solutions for use in, 529
  the application of, in dental practice, 525
Ellerbeck, William Leon, instrumental precision in dental practice, 1
  the application of electro-metallurgy in dental practice, 525
Enamel, relative effect of a common environment upon, 293
Environment, predisposition and, 85
  how governed, 154
Equivalents, foreign, report concerning, 780

Ethyl chloride for obtunding sensitive dentine, 645
Eucalypto-percha, 44

F.

Facing, a fractured porcelain, to repair on a fixed crown or bridge, 434
Facings, making removable, method of, 728
"Faculties," the *Dental Digest* and the, 595
Fetid breath, 670
Fifth District Dental Society, 292
Filling-material, the best, for the temporary teeth, 232
Fillings, to secure good edges to, when using a matrix, 730
Fletcher, M. H., the tongue as a breeding-place for bacteria, 832
Flux, a fluid, that does not pit, 436
  a liquid, 238
Foot-warmer for patients, 192
Foreign countries, a report on the dental schools and laws of, 781
  CORRESPONDENCE: An open letter, 664
  The American Dental Society of Europe, 899
  Relations Committee, 67, 355
    communications from, 286
    report of, 768
Formaldehyde gas for disinfecting books in libraries, 285
Formalin, properties of, 422
Furnaces, home-made porcelain inlay, 729
Fusible metal for articulating models, 517

G.

Galveston Dental Society, 71
Gelatin a hæmostatic, 101
Geranium-formol, after-results of treatment of caries by, 26
Gerhart, Dr. Henry M., obituary, 201
Gingivitis, interstitial, as a prominent obvious early symptom of autointoxication and drug poisoning, 746
  simple, its etiology and treatment, 836
  syphilitic interstitial, 84
Glutol, 653
Gold blindness, or retinal asthenopia, and its treatment, 233
Gold, experiments upon, as a filling material, 9
Growths, malignant, in the mouth, incidental to general practice, 373
Gums, free bleeding of, indicates pyorrhœal conditions, 37
Gutta-percha, setting bridges with, instead of cement, 729

H.

Hæmostatic gelatin, a, 101
Hall, Dr. Horatio Gates, obituary of, 604
Hamilton, Harry F. Some New Appliances, 377
Handles, instrument, 712
  discussion on, 715
Hart, Arch Coombs, Ph.B., D.D.S. M.D., obituary of, 723
Harvard Dental Alumni Association, 733
  Odontological Society, 211
Hatch, Henry D., military dental practice: its modifications and limitations, 749
Hemorrhage, suprarenal capsules in, 324
Heyke, John E., an interesting regulating case, 224
Howe, Dr. J. Morgan, reply to, 65
Hunter, R. W., a few cases of replantation, 313
Hygiene, dental, stray thoughts on, 22
  in schools, 24
Hypertrophy of upper and lower jaws, 390

I.

Illinois State Dental Society, 372, 671
Inlays, 285
 method of obtaining contours of, 106
 suggestions for overcoming difficulties in, 390
 solid gold, 411
 to hold, while cutting the groove, 436
Institute of Dental Pedagogics, 814
International Dental Congress, impressions of the, 218
 plan and scope of, during World's Fair, 1903, 839
 Dental Federation, 789
 first general meeting, held at Cambridge, England, 1901, 868
Investigation, original, not a necessary quality of the teacher, 146
Iodoform, process of manufacture of, 674
Irregularity, case of, of superior cuspid, 17
 discussion on, 32
*Items of Interest*, Reply to, 203

J.

Jaw, hypertrophy of upper and lower, 390
 lower, operation in cases of abnormal protrusion of, 267
Jefferson County Dental Society, 212
Johnson, G. E., the condition of the teeth of children in the public schools, 445

K.

Kiernan, James G., periods of stress and their dental marks, 680
Kowarska's paste, 435

L.

Latham, Vida A., the literature of the pulp, 612
Le Caudey, Dr. Theodore Emanuel, Resolutions of Respect to, 602
Leucoplakia, 392
Libbey, J. A., license: a, diploma; b, examination, 491
License: a, diploma; b, examination, 491

M.

McKellops, Dr. Henry J., obituary of, 437
 Resolutions of Respect to, 439, 666
McNamara, M. C., D.D.S., obituary of, 725
Malocclusions, Treatment of, of the Teeth and Fractures of the Maxilla, review of, 62
Marriage, early and late, social consanguinity, near kin, 296
Massachusetts Dental Society, 441
Mastication, force required in, 311
Materia Medica and Therapeutics, A Practical Treatise on, 125
Matrix, an improvised, 519
 to secure good edges to gold fillings when using a, 730
Maxfield, George A., malignant growths in the mouth, incidental to general practice, 373
Mead, G. N. P., a case of swallowing a tooth, 380
Medical education of dentists, an argument for, 149
 students, instruction of, in the principles of dentistry, 139
Medicine of the nineteenth century, 163
 preventive, oral antisepsis in relation to, 687
Metals, the influence of, in the development of micro-organisms in gelatin, 753
Micro-organisms, non-specificity of, in dental decay, 317
 the influence of metals in the development of, in gelatin, 753

Mills, G. Alden, predisposition and environment, 85
predisposition and environment: how can they be governed? 154
Milwaukee, the conventions at, 655
MISCELLANY:
A banded Logan crown, 284
A durable cement, 351
A fluid flux that does not pit, 436
Alcohol as an antiseptic, 434
Aluminum versus gold for clasps, 435
A method of securing dam clasps on conically shaped teeth, 813
A new method of making dies, 351
of making dummies, 433
An improvised matrix, 519
Cocaine solution for hypersensitive dentine, 731
Curing a boil, 812
Fetid breath, 670
Formaldehyde gas for disinfecting books in libraries, 285
Fusible metal in articulating models, 517
Gold blindness, or retinal asthenopia, and its treatment, 284
Home-made porcelain inlay furnaces, 729
How to lighten full upper cases with side-blocks, 432
Inlays, 285
Kowarska's paste, 435
Local anæsthesia without risk or danger, 812
Method of making removable facings, 728
of saving a badly decayed or fractured root, 435
Microbic association, 520
Miller, Professor W. D., 802
Mounting crowns and bridges, 728
MISCELLANY:
Oxyphosphate of copper, 351
Possibly a valuable drug, 433
Pyorrhœa alveolaris in dogs, 669
Refitting dentures with oxyphosphate of copper, 520
Replacing old rubber for new in rubber and celluloid dentures, 517
Setting bridges with gutta-percha instead of cement, 729
Simple electro-gold-plating apparatus, 730
Testing for disease of the antrum with a tuning-fork, 518
The teeth in pregnancy, 516
The uric acid diathesis, 433
The use of a Logan crown as piers in bridge-work, 669
To finish aluminum plates, 286
To hold inlays while cutting the groove, 436
To remove teeth from rubber plates, 518
To repair a fractured porcelain facing on a fixed crown or bridge, 434
broken plaster models, 669
To secure good edges to gold fillings when using a matrix, 730
To test beeswax, 727
To wax together a broken vulcanite plate, 813
To wet wheels while grinding teeth, 728
Use of rubber dam in inlay work, 436
Weighting a full lower denture, 432
Mississippi Valley Medical Association, 816
annual meeting of, 672
Models, articulating, fusible metal for, 517
broken plaster, to repair, 669

Molar, impacted third, in a mummy, 323
Morgan, William Henry, M.D., D.D.S., death of, 429
obituary of, 521
Mosquito-malarial fever and quinine, 95
Mouth, malignant growths in, incidental to general practice, 373
Mouth-mirrors, polished metal, 33
Movement, and advance, 192
Mummy, impacted third molar in, 323
Musson, Emma E., development of the maxillary sinus, 213

N.

National Association of Dental Faculties, 768
membership of, 786
Association of Dental Examiners, 443, 524, 732
Dental Association, 524, 735
meeting at Milwaukee, special notice, 523
Nausea and vomiting, the prevention of, during anæsthesia, 637
New England Association of Dental Examiners, 444
New Jersey State Dental Society, 72, 442, 734
New York Institute of Stomatology, 32, 255, 324, 390, 560, 643, 849
Odontological Society, 140
State Dental Society, 210
Nichol, James P., the construction of artificial crowns, 302
Nitrous oxide, death under, 89, 167
North Carolina State Board of Dental Examiners, 443
Northeastern Dental Association, 734
NOTES AND COMMENTS:
Are the colleges responsible for the quacks? 138
NOTES AND COMMENTS:
Instruction of medical students in the principles of dentistry, 139
Notice, 371

O.

OBITUARIES:
Beers, W. George, L.D.S., D.D.S., 135
Chupein, Dr. Theodore F., 352
Gerhart, Dr. Henry M., 201
Hall, Dr. Horatio Gates, 604
Hart, Arch Coombs, Ph.B., D.D.S., M.D., 723
McKellops, Dr. Henry J., 437, 666
McNamara, M. C., D.D.S., 725
Morgan, William Henry, M.D., D.D.S., 521
Saunders, Sir Edwin, F.R.C.S., 440
Williams, Dr. Nelson W., 602
Occlusion, a method of getting, in a case with a shell crown, 340
Onderdonk, T. W., the treatment of unfilled root-canals, 20
Operative Dentistry, The American Text-Book of, review of, 59
Oral cavity, uric acid in its relation to disease, with special reference to the diseases of the, 534
ORIGINAL COMMUNICATIONS:
Address of the Chairman of the Section on Stomatology of American Medical Association, 473
A case of orthodontia diagnosed and progress watched by the X-ray, 141
A case of swallowing a tooth, 380
A few cases of replantation, 313
An argument for medical education of dentists, 149
An interesting regulating case, 224
Bacteriology, 632
Boxings, 73

Original Communications:
Case of irregularity of superior cuspid, 17
Development of the maxillary sinus, 213
Electricity as applied to chemical and metallurgical processes, 673
Force required in mastication, 311
Free use of tooth-brushes essential for healthful preservation of teeth and gums, 225
Impressions of the International Dental Congress, 218
Infectious diseases, 686
Instrumental precision in dental practice, 1
Interstitial gingivitis as a prominent obvious early symptom of autointoxication and drug poisoning, 746
License: a, diploma; b, examination, 491
Malignant growths in the mouth, incidental to general practice, 373
Methods in the preparation of teeth, 625
Military dental practice; its modifications and limitations, 749
Oral antisepsis in relation to preventive medicine, 687
Oral prophylaxis, 817
Original investigation not a necessary quality of the teacher, 146
Periods of stress and their dental marks, 680
Plan and scope of an International Dental Congress during World's Fair, 1903, 839
Predisposition and environment: how can they be governed? 154
Prophylaxis, 693

Original Communications:
Relative effect of a common environment upon enamel, 293
Revenue for conducting the work of the boards of dental examiners, 495, 496
Social consanguinity, near kin, early and late marriage, 296
Simple gingivitis, its etiology and treatment, 836
Some æsthetic considerations in the treatment of teeth in the incisal region, 737
Some considerations of the dental outlook in Cuba, 307
Some new appliances, 377
Stray thoughts on dental hygiene, 22
Symposium on the degeneracy of the pulp, preliminary work, 545
The application of electro-metallurgy in dental practice, 525
The appointment of State boards of medical and dental examiners, 480
The condition of the teeth of children in the public schools, 445
The construction of artificial crowns, 302
The dental college standard, 485
The duty of young men to the profession, 150
The literature of the pulp, 612
The tongue as a breeding-place for bacteria, 832
The treatment of certain oral expressions commonly designated pyorrhœa alveolaris, 605
The treatment of unfilled root-canals, 20
The use of an assistant, 75

Original Communications:
Tin: a plea for more conservative methods in filling teeth, 461
Unilateral jumping the bite, 315
Uric acid in its relation to disease, with special reference to the diseases of the oral cavity, 534
Use of silver-foil in dentistry, 690
Orthodontia, a case of, diagnosed and progress watched by the X-ray, 141
Oxyphosphate of copper, 351
refitting dentures with, 520

P.

Palate, congenital cleft, 263
Pan-American Medical Congress, The Third, 69
Parmele, George L., revenue for conducting the work of State boards of dental examiners, 496
Pennsylvania Board of Dental Examiners, 211, 441
State Dental Society, 815
Pierce, C. N., bacteriology, 632
Plaques, 50
gelatinous microbic, 476
Plastic fillings, scrapers for finishing, 561
Plates, aluminum, to finish, 286
broken vulcanite, to wax together, 813
rubber, to remove teeth from, 518
Poisoning, drug, interstitial gingivitis as a prominent obvious early symptom of, 746
Polishing a stimulus to better tooth-building, 48
wheels, made of corundum and gelatin, 423
Porcelain, cracking of, in soldering, 238
Porcelain, Dental Art, A Practical Treatise on, 128
silicate of, 344
Practice, incidents of, 767
military dental; its modifications and limitations, 749
success in, much bothering about a non-essential for, 78
Precision, instrumental, in dental practice, 1
Predisposition and environment, 85
how can they be governed? 154
Pregnancy, the teeth in, 516
Pressure-anæsthesia, immediate removal of pulps by, 421
Profession, the duty of young men to the, 150
Prophylaxis, 693
in dentistry, vol. xxi., 805
discussion on, 44, 234
oral, 817
Public schools, the condition of the teeth of the children in, 445
Pulp anæsthetized by injections of chloroform and carbolic acid, 424
by a solution of hydrochlorate of cocaine and pressure, 425
bibliography of the literature on, 623
blood-supply of, 616
capping the, 386
immediate removal of, by pressure anæsthesia, 421
nerve-supply of, 618
removal of, by cocaine and pressure, 255
symposium on the degeneracy of the, preliminary work, 545
the literature of, 612
Pulp-nodules, the development of, 697
Pyorrhœa alveolaris, four classes of, 648
in dogs, 669

Pyorrhœa alveolaris, produced by mechanical irritation, 247
the treatment of certain oral expressions commonly designated, 605
under prophylactic treatment, 238
Pyorrhœal conditions indicated by freely bleeding gums, 27

Q.

Quack dentistry, 502
discussion on, 504
press favors, 505
remedy for, 505, 509
Quacks, are the colleges responsible for the, 138
Quinine, the mosquito-malarial fever and, 95

R.

Regulating Case, An Interesting, 224
Replantation, a few cases in, 313
Reply to Dr. J. Morgan Howe, 65
Report of the Foreign Relations Committee, 206
Report of Society Meetings:
Academy of Stomatology, 44, 111, 168, 264, 336, 420, 502, 711
American Academy of Dental Science, 52, 185, 233, 407, 570, 755
American Medical Association, Section on Stomatology, 473, 549, 639, 702, 856
Resolutions upon the appointment of Dr. John S. Marshall as examiner, 560
International Dental Federation, 789
first general meeting, held at Cambridge, England, 1901, 868
Report of Society Meetings:
National Association of Dental Faculties, 768
New York Institute of Stomatology, 32, 102, 255, 324, 390, 560, 643, 849
Fund for Scientific Research, 568
Requirements, Minimum, Report concerning, 784
Resolutions of Respect to Dr. H. J. McKellops, 439
Dr. Theodore F. Chupein, 603, 726
Dr. Theodore Emanuel Le Caudey, 602
Review of Dental Literature:
A Contribution to the Sterilization of Dentine in Living and Dead Teeth, 841
Actinomycosis, 231
An Attempt to Explain the Sensitiveness of Dentine, 321
A Treatment of Pulpless Teeth, 231
Capping the Pulp, 386
Carbonic Acid in Dental Practice, 388
Chloretone as a Hypnotic and Local Anæsthetic, 383
Gold Blindness, or Retinal Asthenopia, and its Treatment, 233
Impacted Third Molar in a Mummy, 323
Syphilitic Localosis Alveolaris, 232
The Best Filling-Material for the Temporary Teeth, 232
The Demands for Tooth Hygiene, 500
The Development of Pulp-Nodules, 697
The Influence of Metals in the Development of Micro-Organisms in Gelatin, 753
The Makers of Dentistry, 755

REVIEW OF DENTAL LITERATURE:
The Non-Specificity of Micro-Organisms in Dental Decay, 317
discussion on, 317
The Prevention of Nausea and Vomiting during Anæsthesia, 637
The Saliva, a Protection afforded by Nature against Caries, 696
Rhode Island Dental Society, Resolution, 140
Rochester Dental Society, Programme of, sessions 1900-1901, 72
Root, badly decayed or fractured, method of saving, 435
Root-canals, capillarity in treatment of, 40
filling, with oxyphosphate with a syringe, 645
unfilled, classification of, 20
the treatment of, 20
Root-filling, a process of immediate, 420
Rubber dam, use of, in inlay work, 436

S.

Saliva, a protection afforded by nature against caries, 696
comments on Dr. Michael's paper on, 223
human, investigations of, 476
Saunders, Sir Edwin, obituary of, 440
Schools, foreign dental, 776
Scraper for finishing plastic fillings, 561
Short-necked persons, a dangerous class under nitrous oxide, 89
Shumway, T. D., tin: a plea for more conservative methods in filling teeth, 461
Side-blocks, how to lighten full upper cases with, 432
Silking out between the teeth, 48
Silver, chloride of, a solder for aluminum, 676
Silver-foil, use of, in dentistry, 690
Sinus, the maxillary, development of, 213
Smith, B. Holly, some æsthetic considerations in the treatment of teeth in the incisal region, 737
Smith, D. D., Oral prophylaxis, 817
Solutions, depositing, for use in electro-metallurgy, 529
management of plating, 531
South Dakota State Dental Society, 444
Southern California Dental Association, 734
Southern Dental Society of New Jersey, 211
Spinal anæsthesia, 93
injections for, how made, 94
Standard, the dental college, 485
State boards of dental examiners, 475
boards of medical and dental examiners, the appointment of, 480
dental examining boards, discussion on, 111, 549
dental examining boards and their questions, vol. xxi., 784
discussion on, 111
Steeves, Alice M., infectious diseases, 686
Sterilizing instruments, an effective method of, 105
Stick-holder for use in cleaning teeth, 257
Stockwell, C. T., New Modes of Thought based upon the New Materialism and the New Pantheism, 897
Stress, periods of, and their dental marks, 680
Suprarenal capsules in hemorrhage, 324
discussion on, 325
Surgeons, dental, in the army, 197

Syphilis, microbe of, 562
Syringe, a French, 424
for filling root-canals with oxyphosphate, 645

T.

Talbot, Eugene S., an argument for medical education of dentists, 149
interstitial gingivitis as a prominent obvious early symptom of autointoxication and drug poisoning, 746
social consanguinity, near kin, early and late marriage, 296
symposium on the degeneracy of the pulp, preliminary work, 545
syphilitic interstitial gingivitis, 84
unilateral jumping the bite, 315
Teacher, original investigation not a necessary quality of the, 146
Teaching, the essentials in, 193
Teeth and gums, free use of tooth-brushes essential for healthful preservation of, 225
bibliography of the methods in the preparation of the, 631
cleaning, stick-holder for use in, 257
clean, will not decay, 335
filling, a plea for more conservative methods in, 461
in pregnancy, 516
lower front, a unique method of tightening or inserting, 248
discussion, 253
methods in the preparation of, 625
pulpless, a treatment of, 231
silking out between, 48
Teeth and gums, some æsthetic considerations in the treatment of, in the incisal regions, 737
temporary, the best filling-material for, 232
the condition of, of children in the public schools, 445
to remove, from rubber plates, 518
Tennessee Dental Association, 442
Texas State Dental Association, 670
The convention period, editorial, 510
The editor was tired, editorial, 121
Theory and practice, communications on, 102, 255, 324, 390, 560, 643
The twentieth century, editorial, 55
Thomas, Frank McS., force required in mastication, 311
Thorpe, Burton L., Plan and scope of an International Dental Congress during World's Fair, 1903, 839
Tin, fillings of, with gold burnished on the surface, 103
Tongue, the, as a breeding-place for bacteria, 832
cancer of, the distinguishing features of, 392
Tooth, a case of swallowing a, 380
Tooth-brushes, free use of, essential for healthful preservation of teeth and gums, 225
Tooth-building, better, polishing a stimulus to, 48
Tooth hygiene, the demands for, 500
Toxins, nature and use of, 401
Trade, wherein does dentistry differ from, 796
Tri-State Dental Association, 292, 372
a legal notice, 209
Tuning-fork, testing for disease of the antrum with a, 518
Turner, V. E., revenue for conducting the work of boards of dental examiners, 495

U.

Union meeting of the Sixth, Seventh, and Eighth District Dental Societies of New York, 735

Uric acid in its relation to the diseases of the oral cavity, 534

V.

Vela, method of constructing, for adult and adolescent cleft palate, 222

Volume, a new, the beginning of, editorial, 57

W.

War Department, circular from, 282

Watkins, J. Conard, electricity as applied to chemical and metallurgical processes, 673

Wheels, to wet, while grinding teeth, 728

Williams, Dr. Nelson W., obituary of, 602

Williams, E. Lloyd, a method of constructing vela for adult and adolescent cleft palate, 222

Wilson, Erastus, some considerations of the dental outlook in Cuba, 307

Wright, C. M., oral antisepsis in relation to preventive medicine, 687
  original investigation not a necessary quality of the teacher, 146

X.

X-ray, a case of orthodontia diagnosed and watched by the, 141

Y.

Young men, the duty of, to the profession, 150

